# Genome sequence of *Methylobacterium fujisawaense* strain C14, isolated from concrete

**DOI:** 10.1128/mra.00531-24

**Published:** 2024-07-02

**Authors:** E. Anders Kiledal, Iz Moxley, Olga Shevchenko, Jennifer L. Goff, Julia A. Maresca

**Affiliations:** 1 Department of Biological Sciences, University of Delaware, Newark, Delaware, USA; 2 Department of Chemistry, SUNY College of Environmental Science and Forestry, Syracuse, New York, USA; 3 Sequencing and Genotyping Center, University of Delaware, Newark, Delaware, USA; California State University San Marcos, San Marcos, California, USA

**Keywords:** concrete, built environment, *Methylobacteria*, genome editing, plasmids

## Abstract

*Methylobacterium fujisawaense* strain C14 was isolated from a weathered concrete cylinder. Using PacBio sequencing, we generated a complete genome for strain C14, which includes one circular chromosome (6,656,731 bp) and six putative plasmids (35,452 to 85,428 bp).

## ANNOUNCEMENT

Strain C14 was isolated from concrete prepared using materials and a mix design from the Delaware Department of Transportation and weathered outside at the University of Delaware (39°40'48.2"N 75°45'00.0"W) from 2 May 2013 to 17 August 2014 (1). As other *Methylobacterium* spp. are genetically tractable (2), we sequenced the C14 genome to facilitate tool development for engineering isolates from concrete.

The concrete cylinder was cut into ~2 cm slices using an alcohol-sterilized saw blade. The slices were broken with a hammer. Small (<2 cm^3^) pieces were placed in 30 mL phosphate-buffered saline (137 mM NaCl, 2.7 mM KCl, 8 mM Na_2_HPO_4_, 2 mM KH_2_PO_4_) with 0.5% TWEEN-20, sonicated in an ultrasonic eyeglass cleaner for 5 min, shaken at 25°C at ~180 rpm for 2 h; and then plated on 10% standard BG-11 medium (3) amended with 2.5 g L^−1^ each peptone, yeast extract, and glucose and solidified with 7.5 g L^−1^ gellan gum and 8 mM CaCl_2_. Plates were incubated at 28°C for ~2 weeks. A pink colony was re-streaked on the same medium 4 times and archived in 10% glycerol at −80°C.

Before sequencing, strain C14 was revived from the glycerol stock on Luria-Bertani plates (LB, Fisher Scientific, catalog # DF0446-17-3) and incubated at room temperature until colonies appeared. A single colony was inoculated into 10 mL LB and incubated with shaking at 25°C until the stationary phase. DNA was extracted using phenol-chloroform extraction (4). A single-molecule real-time (SMRT) library was barcoded and prepared using the PacBio SMRTbell Express template preparation kit (v3.0). DNA was sheared to 12 kb using a Megaruptor3 (Diagenode), and fragments >6 kb were size-selected using BluePippin (Sage Science). The average library fragment size was 12 kb, as measured by a fragment analyzer (Advanced Analytical Technologies, Inc.). Sequencing was completed on a PacBio Sequel IIe single-molecule sequencer in one 8M SMRT Cell with 30-h movie. Samples were demultiplexed using PacBio SMRT Link version 11. The PacBio sequencing yielded 55,332 raw reads with an N50 of 12,235 bp.

The genome was assembled and annotated using KBase (5). Filtlong v0.2.1 [*kb_flye v0.2.2* (6)] was used to filter reads <1000 bp, leaving 35,425 reads. The reads were assembled, polished, and circularized with Flye v2.9.2 [*kb_flye v0.2.2* (7)]. Genome completeness and contamination were estimated at 100.0% and 0.0%, respectively, using CheckM v1.0.18 [*kb_Msuite v1.4.2* (8)]. Mobile genetic elements were predicted using geNomad v1.7.5 (9) implemented in NMDC-EDGE (10). The genome was annotated using RASTtk v1.073 [*RAST_SDK v1.9.5* (11)], available in the accompanying KBase narrative (12). The assembled genome was deposited in GenBank and reannotated using the Prokaryotic Genome Annotation Pipeline v6.7 (13). Default parameters were used except where noted.

The assembled strain C14 genome contains 7,094,668 bp across nine contigs, with an average GC content of 69.3% (Table 1). Contig_1 is the 6,656,731 bp chromosome. Based on the geNomad results, contig_3 is a predicted extrachromosomal phage, and the remaining contigs are predicted plasmids. RASTtk and PGAP annotated 7,378 and 6,709 CDS, respectively. The GTDB-Tk v1.7.0 [*kb_gtdbtk v1.0.0* (14)] utility in KBase identified C14 as a *Methylobacterium fujisawaense* strain (15).

**TABLE 1:** Descriptions of chromosome and putative plasmids^*a*^

Contig	Description	GC (%)	Length (bp)	Topology	Coverage	GenBank accession #
contig_1	Chromosome	69.5	6,656,731	Circular	68X	CP155000
contig_2	Plasmid	65.2	62,136	Circular	87X	CP155001
contig_3	Extrachromosomal phage	66.9	33,746	Circular	51X	CP155002
contig_4	Plasmid	69.3	35,452	Linear	132X	CP155003
contig_5	Plasmid	66.6	51,201	Circular	24X	CP155004
contig_6	Plasmid	66.5	39,253	Linear	21X	CP155005
contig_7	Plasmid	66.5	76,184	Circular	88X	CP155006
contig_8	Plasmid	66.2	85,428	Circular	116X	CP155007
contig_9	Plasmid	65.5	54,537	Circular	88X	CP155008

^*a*^ Descriptions of plasmids and the putative extrachromosomal phage are based on geNomad output. The chromosome and plasmids were circularized in flye v. 2.9.2.

## Data Availability

Raw sequencing data is available at the Short Read Archive with accession number SRR28874887. The assembled and PGAP-annotated chromosome and plasmids are available at NCBI with accession numbers CP155000 (chromosome) and CP155001 to CP155008 (plasmids and extrachromosomal phage). The raw reads, genome, and RASTtk annotations are available in the KBase narrative https://kbase.us/n/169030/43/ (13).
